# Modulation of Vitamin D Status and Dietary Calcium Affects Bone Mineral Density and Mineral Metabolism in Göttingen Minipigs

**DOI:** 10.1155/2013/460512

**Published:** 2013-08-24

**Authors:** Katharina E. Scholz-Ahrens, Claus-Christian Glüer, Felix Bronner, Günter Delling, Yahya Açil, Hans-Jürgen Hahne, Joachim Hassenpflug, Wolfram Timm, Jürgen Schrezenmeir

**Affiliations:** ^1^Institute of Physiology and Biochemistry of Nutrition, Max Rubner-Institut (MRI), Federal Research Institute of Nutrition and Food-Kiel, 24103 Kiel, Germany; ^2^Department of Safety and Quality of Milk and Fish Products, Max Rubner-Institut (MRI), Federal Research Institute of Nutrition and Food-Kiel, 24103 Kiel, Germany; ^3^Medical Physics Research Group, Department of Diagnostic Radiology, University Hospital Schleswig-Holstein, Campus Kiel, 24105 Kiel, Germany; ^4^The University of Connecticut Health Center, Farmington, CT 06030, USA; ^5^Department of Osteopathology, University Medical Center Hamburg-Eppendorf, 20246 Hamburg, Germany; ^6^Department of Oral and Maxillofacial Surgery, University Hospital Schleswig-Holstein, Campus Kiel, 24105 Kiel, Germany; ^7^Department of Orthopaedics, University Hospital Schleswig-Holstein, Campus Kiel, 24105 Kiel, Germany

## Abstract

Calcium and vitamin D deficiency impairs bone health and may cause rickets in children and osteomalacia in adults. Large animal models are useful to study experimental osteopathies and associated metabolic changes. We intended to modulate vitamin D status and induce nutritional osteomalacia in minipigs. The control group (*n* = 9) was fed a semisynthetic reference diet with 6 g calcium and 6,500 IU vitamin D_3_/kg and the experimental group (*n* = 10) the same diet but with only 2 g calcium/kg and without vitamin D. After 15 months, the deficient animals were in negative calcium balance, having lost bone mineral density significantly (means ± SEM) with −51.2 ± 14.7 mg/cm^3^ in contrast to controls (−2.3 ± 11.8 mg/cm^3^), whose calcium balance remained positive. Their osteoid surface was significantly higher, typical of osteomalacia. Their plasma 25(OH)D dropped significantly from 60.1 ± 11.4 nmol/L to 15.3 ± 3.4 nmol/L within 10 months, whereas that of the control group on the reference diet rose. Urinary phosphorus excretion and plasma 1,25-dihydroxyvitamin D concentrations were significantly higher and final plasma calcium significantly lower than in controls. We conclude that the minipig is a promising large animal model to induce nutritional osteomalacia and to study the time course of hypovitaminosis D and associated functional effects.

## 1. Introduction

Adequate calcium and vitamin D intakes are essential for skeletal health and to minimize the incidence of osteoporosis and of rickets in children and nutritional osteomalacia in adults. Calcium and vitamin D supplementation is also key to the treatment of these diseases [1–3]. Rickets and osteomalacia remain endemic problems in developing countries but show increasing prevalence also in North America and Europe [3, 4] because of changes in life style with an increase of indoor activities and a decrease in sunlight exposure. Incidence of osteomalacia is also increased by the demographic shift, because calcium and vitamin D intakes are often inadequate in the elderly, in whom vitamin D synthesis, calcium absorption, and renal reabsorption tend to diminish [3]. Subjects with darker skin or covering clothing habits are at higher risk because both factors contribute to insufficient dermal vitamin D synthesis, responsible in turn for diminished bone density. Vitamin D combined with calcium has been shown to enhance lower-extremity function when vitamin D intake was sufficient to assure a serum concentration of 25-hydroxyvitamin D [25(OH)D] that exceeds 75 nmol/L [5]. This value is much higher than the current normative value of 25 nmol/L, on the basis of which the incidence of rickets/osteomalacia is minimized [1]. Calcium and vitamin D thus have additional metabolic functions, with vitamin D insufficiency now recognized to be associated with several chronic diseases [5, 6]. How much vitamin D is needed to achieve an adequate value for plasma 25(OH)D is not well known, because the estimate of optimal vitamin D intake is complicated by several confounding factors that affect vitamin D status, beside age, diet, and sunlight exposure. Obviously there is no final consensus on the definition of hypovitaminosis, vitamin D deficiency or insufficiency and on what an optimal 25(OH)D level is [1, 5, 7]. 

It is still difficult to distinguish osteoporotic changes from those of nutritional osteomalacia solely by noninvasive, radiological techniques (quantitative computed tomography, QCT). The loss of apparent bone mineral density (BMD) following rarefaction of trabecular bone, which often is amplified by a calcium and vitamin D shortage, like in osteoporosis, cannot be differentiated from a loss following impaired mineralization of bone tissue, like in moderate osteomalacia. The characteristic feature of nutritional osteomalacia is low bone matrix mineralization combined with enlarged thickness of osteoid tissue in the presence of chronic and severe vitamin D deficiency [1, 8, 9]. 

Enlarged osteoid has to be verified histologically on bone biopsies as surface or volume [8, 9]. To facilitate differentiation of osteomalacia from other low-BMD diseases, like osteoporosis, changes in functional parameters of bone and mineral metabolism may be assessed by use of laboratory markers. Studying the time course of BMD and serum levels of 25(OH)D and 1,25(OH)_2_D in human subjects in response to varying calcium and vitamin D intakes requires an extensive and expensive experimental set-up and is limited by ethical issues. Investigations in animal models may help in this respect and provide a model for preclinical trials with agents modulating vitamin D metabolism and vitamin D actions. Experiments with rodents have yielded useful insights, but guidelines application to human therapy of bone diseases, like for osteoporosis, may require studies with larger animal models [10]. In previous publications [11–13] we reported on the utilization of the minipig as a large animal model in metabolic and bone studies including glucocorticoid-induced osteoporosis (GIO). We now report on the long-term effects of vitamin D and calcium insufficiency on longitudinal and cross-sectional changes of bone mineral density and content, osteoid surface and parameters of bone metabolism compared to a reference group on sufficient intake of calcium and vitamin D. We aimed at assessing whether the minipig can be used for the study of nutritional osteomalacia and the modeling of vitamin D status.

## 2. Experimental Methods

The experiment was approved by the *Ministerium für Umwelt*, *Natur und Forsten des Landes Schleswig-Holstein* that regulates animal experiments. The experiment was part of a larger intervention trial with minipigs, in which we investigated the physiologic responses and the composition and properties of bone in this large animal model to various treatments [11, 13]. 

### 2.1. Animals, Experimental Groups, and Time Schedule

The experiment was initiated with two groups of ten female primiparous Göttingen minipigs, 30 months old, from the Institute's own breeding herd. They were matched for age and body weight. Blood, urine, and fecal samples were obtained, and BMD was determined at the beginning of the experiment and at regular intervals thereafter. Animals were sacrificed after 15 months, when bone specimens were obtained. One animal died during anaesthesia. 

### 2.2. Diets, Housing, and Intervention

Until the start of the study, the sows were on the Institute's regular feeding regimen for minipigs (standard diet, 580 g/d) that provided per kg diet: 5 g calcium, 4 g phosphorus, 50 *μ*g (2000 IU) cholecalciferol (vitamin D_3_), and 9 MJ metabolizable energy. Accordingly, the vitamin D intake was 29 *μ*g/d (1,160 IU/d) before intervention. The sows were then assigned either to the control group [C] or to the deficient group [CaD(−)], getting 370 g/d of a semipurified diet. The control group was fed a reference diet that contained (g/kg diet) corn starch, 290; sucrose, 240; casein, 150; cellulose, 80; lard, 75; margarine, 75; mineral and vitamin premix, 60; lactulose, 30; and 14 MJ metabolizable energy. The diet contained 6 g/kg calcium, 7 g/kg phosphorus, and 160 *μ*g/kg (6,500 IU/kg) of vitamin D_3_ (Deutsche Vilomix Tierernährung GmbH, Neuenkirchen-Vörden, Germany). Their vitamin D intake was 60 *μ*g/d (2,400 IU/d). The reference diet was different from a standard diet for pigs because we aimed to mimic a typical western diet (i.e., energy dense, rich in fat and protein, poor in fiber, Ca : P < 1), but with mineral and vitamin content sufficient for adult minipigs. This reference diet has been long used in our laboratory for experimental studies without any signs of side effects and was described before [14]. Its vitamin D content is higher than the standard diet for pigs or values recommended for humans, owing to the matter that less feed was supplied because of the higher energy density of the semisynthetic diet. Furthermore, the experiment was long-termed and without other oral or dermal vitamin D sources. The vitamin D dose remained below the upper tolerable level for humans of 4000 IU per day and assumed toxicity levels of 10,000 IU/day [1]. The diet was analyzed for protein, calcium, and phosphorus. Animals on the deficient diet were fed the same semisynthetic diet except that it contained only 2 g/kg calcium and no vitamin D_3_. For maintenance of body weights individual feed intake was restricted to 370 g/d, all of which was consumed by all animals. Deionized water was available *ad libitum*. Animals were housed under controlled conditions on strawless floor cages in individual pens without daylight but artificial light between 06:00 h and 18:00 h, temperature between 20 and 22°C, and 60–70% humidity. At the end of the experiment, animals were anaesthetized with a combination of Midazolam, (1 mg/kg body weight) and ketamine hydrochloride (5 mg/kg body weight; CuraMed Pharma, Karlsruhe, Germany) and sacrificed by exsanguination. Representative bone specimens were stored at −18°C.

### 2.3. Biochemical Parameters

Blood samples were collected between 08:00 and 10:00 after an overnight fast at *t*0 and after 2, 5, 7, 10, 13, and 15 months (*t*2 to *t*15). Calcium content was analyzed in EDTA plasma by atomic absorption spectroscopy (Perkin Elmer 1100) with air/acetylene at 2300°C. Phosphorus was determined in EDTA plasma as inorganic phosphorus with an automatic analyzer (Cobas Bio, Hoffmann La Roche, Basel, Switzerland) and a test kit (Roche Diagnostics GmbH, Mannheim, Germany). PTH was determined in EDTA plasma using a human radioimmunoassay (RIA) (Immundiagnostik, Bensheim, Germany) according to [15]. The kit contained a goat antiserum directed against the C-terminal end of PTH (53–84), with a donkey-anti-goat immunoglobulin as precipitation reagent and a synthetic human PTH fragment as a standard. Values for sensitivity, intra- and interassay variability were 3.4 pg/L, 9.2% coefficient of variation (CV) and 12.5% CV, respectively. 25-Hydroxyvitamin D [25(OH)D] was measured in EDTA plasma by RIA (Immundiagnostik, Bensheim, Germany) after acetonitrile precipitation. Values for sensitivity, intra- and interassay variability were 2.5 nmol/L, 9.8% CV and 14% CV, respectively. 1,25-Dihydroxyvitamin D_3_ [1,25(OH)_2_D] was measured in EDTA plasma by radio-receptor assay (RRA) (Immundiagnostik, Bensheim, Germany). Values for intra- and interassay variability were 12% CV and 17% CV, respectively. Osteoprotegerin (OPG) was analyzed in serum by sandwich enzyme immunoassay (Immundiagnostik, Bensheim, Germany) in the samples at baseline and after eight months. Values for intra- and interassay variability were 9% CV and <10% CV, respectively, at a detection limit of 0.14 pmol/L. Bone alkaline phosphatase (BAP) was analyzed in heparin plasma as the difference between total alkaline phosphatase (AP) before and after lectin precipitation, with a kit (Roche Diagnostics GmbH, Mannheim, Germany) based on a kinetic color reaction. Values for intra- and interassay variability were 1.6% CV and 2.5% CV, respectively. Urine was collected for 24 h at *t*0 and *t*2 to *t*15 and stored frozen at −18°C until analysis. For this, minipigs were individually transferred to metabolic cages for 24 h that allowed the separate sampling of urine and feces. For technical reasons, urine samples of only 6 animals per group were collected at *t*2. Calcium and phosphorus were analyzed as described previously. Deoxypyridinoline (DPD) was determined by a preparative reverse-phase-column HPLC as described before [16], using a commercially available bovine bone gelatin (Deutsche Gelatine-Fabriken Baden, Germany) as external standards. All plasma and urine parameters analyses were done in duplicate and blindly.

### 2.4. Mineral Balance

Metabolic balances were performed at *t*0, *t*5, *t*10, and *t*15. Minipigs were transferred to metabolic cages for 7 days to allow separation of feces and urine. Samples were collected daily and stored at −18°C until analysis. Calcium and phosphorus absorption and retention were calculated on a mg/7-day basis as follows: absorption = intake − fecal excretion; retention = intake − (fecal excretion + urinary excretion). All analyses were done in duplicate and blindly. 

### 2.5. Bone Mineral Density

(BMD) was assessed *in vivo* in aneasthetized animals by quantitative computed tomography (QCT) with a Siemens Sensation 16 Scanner at *t*0, *t*8, and *t*15. Trabecular BMD values were evaluated in a central bone slice, 10 mm thick, and an elliptical region in the anterior part of lumbar vertebrae L1–L3. The Siemens OsteoPackage was used to calculate results. Reproducibility was assessed by duplicate measurements in 4 minipigs with interim repositioning. The root-mean-square (RMS) average of precision [17] was 0.83% (3.4 mg/cm^3^).

*Bone strength* was measured on the 4th lumbar vertebra (L4) as ultimate mechanical stress and calculated as failure load, divided by the cross-sectional area of the cylinders at the point of failure. Compression was tested on thawed cylindrical cancellous bone specimens with a diameter of 7.5 mm and 10 mm in length. This had been harvested from the centre of the vertebral bodies according to [18] with the aid of a core drill and a parallel saw. Lumbar vertebrae were fixed and centralized in a bench vise when the drilling was performed from the cover plate to the ground plate under visual inspection to ensure that these specimens consisted solely of trabecular bone. Compression was tested in the Sensotec (Columbus, Ohio) device and deformation in the MTS System (Berlin, Germany). Ultimate strain was measured at the point of failure. Young's modulus was calculated from the slope of the linear portion of the regression of the stress-strain relationship.

### 2.6. Bone Chemical Composition

Ash, calcium, and phosphorus content were determined on the same crushed cylindrical sample of the 4th lumbar vertebra (L4) after mechanical testing for bone strength. Samples were dried for 4 h at 105°C to determine dry matter (%). The dried bone was ashed overnight in a muffle furnace at 450°C; the ash was weighed, dissolved in 20% (v/v) HCl. Calcium and inorganic phosphorus were analyzed as described previously. Bone fat content was determined on a cylindrical sample of exclusively trabecular bone of the last breast vertebra. The sample was ground, hydrolyzed with 4 M HCl, and extracted with petrol ether [19]. Analyses were done in duplicate and blindly.

### 2.7. Histomorphometry

The 2nd lumbar vertebrae were used for histomorphometry [20] and evaluated for bone volume/tissue volume (BV/TV), trabecular width (Tb.Wi), trabecular separation (Tb.Sp), osteoid volume/tissue volume (OV/TV), osteoid surface/bone surface (OS/BS), osteoblast surface/bone surface (Ob.S/BS), eroded surface/bone surface (ES/BS), osteoclast surface/bone surface (Oc.S/BS), number of osteoclasts/bone surface (N.Oc/BS), and number of osteoclasts/tissue area (N.Oc/T.Ar).

### 2.8. Statistical Analysis

Due to the limited number of animals we performed an explorative statistical approach. The statistical package Statgraphics 4.1 (Manugistics, Inc., Rockville, MD, USA) was used to perform the analysis of variance (ANOVA) and to calculate the mean and standard error of the means (SEM). The parameters were distributed normally or in a nearly normal fashion, and differences between the two experimental groups were evaluated by Student's *t*-test (two-sided) for cross-sectional comparison, except for osteoid surface. For this, a one-sided test was applied according to the hypothesis that vitamin D deficiency induces enlarged osteoid. The longitudinal data within groups either as changes from baseline or absolute values were tested by the paired *t*-test at each time point. *P* ≤ 0.1 is characterized as a trend, and *P* ≤ 0.05 was taken as statistically significant.

## 3. Results

Age, body weight, and characteristics of calcium and bone metabolism at baseline did not differ significantly between the groups (Tables 1–3). Animals on the deficient diet tended to lose weight by −1.4% after 5 months and by −3.7% after 15 months. Body weights in the control group remained stable (Table 2). 

### 3.1. Osteomalacia

**Figure 1:**
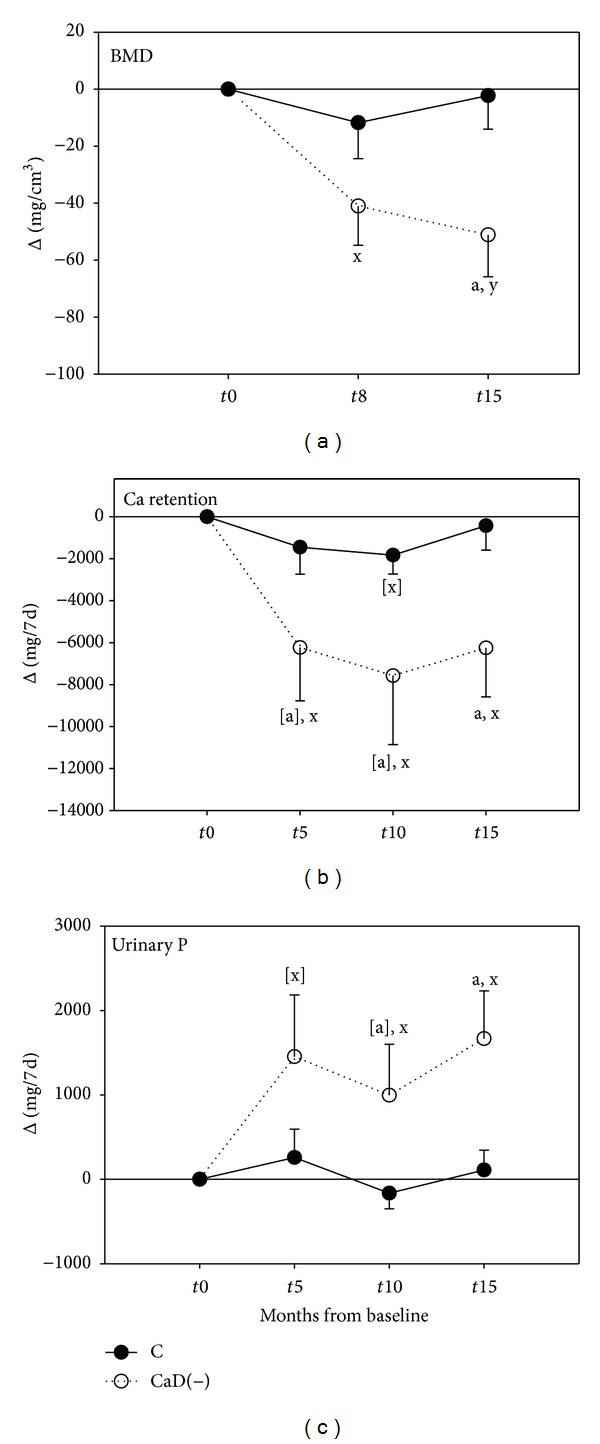
Effects of calcium and vitamin D deficiency [CaD(−); dotted line] on bone mineral density (BMD, (a)), calcium retention (b), and urinary excretion of phosphorus, (c), compared with a control group (C; solid line). Values are changes from baseline *t*0, with baseline values assigned to zero. Means and SEM, *n* = 8–10. Significantly different from the controls at a given time: ^[a]^ *P* < 0.1 (trend); ^a^ *P* ≤ 0.05. Significantly different from baseline: ^[x]^ *P* ≤ 0.1; ^x^ *P* ≤ 0.05; ^y^ *P* ≤ 0.01.

**Figure 2:**
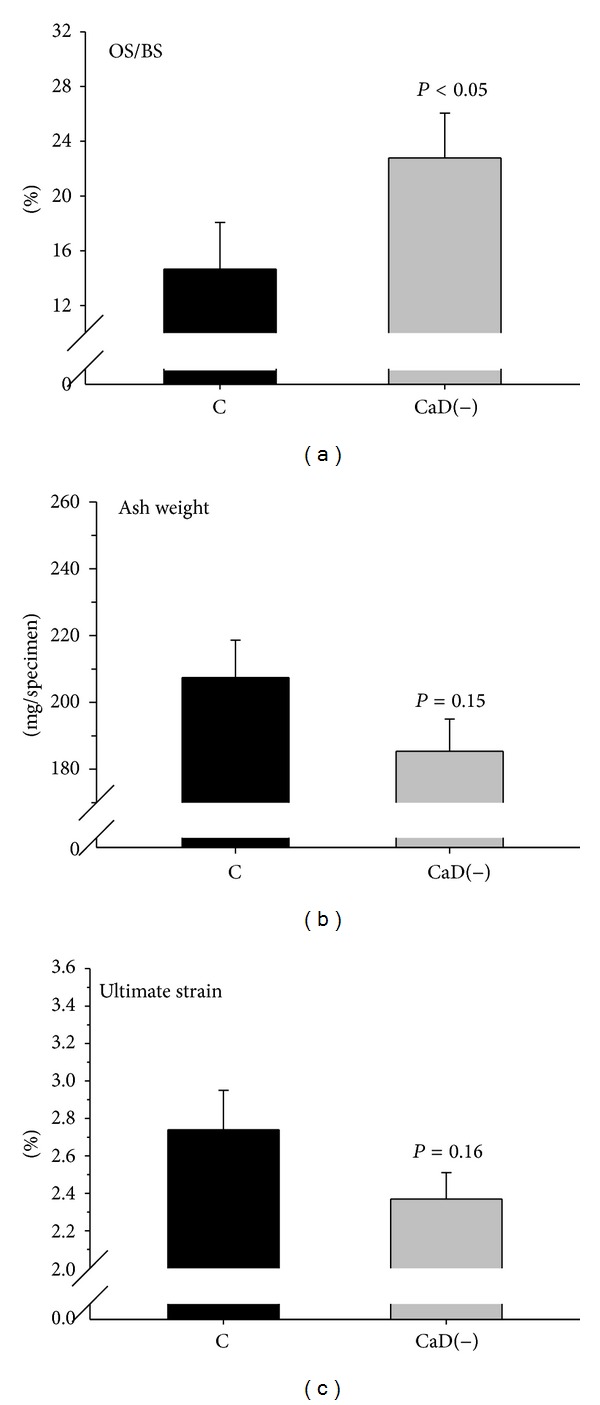
Effects of calcium and vitamin D deficiency [CaD(−)] on osteoid surface [OS/BS], (a), bone ash weight (b), and ultimate mechanical strain (c) compared with a control group [C]. Means and SEM, *n* = 8–10. *P* < 0.05 = significantly different from the control group.

Longitudinally BMD *in vivo* (mg/cm^3^) decreased significantly in animals on the deficient diet (Figure 1(a)). After 15 months, BMD had declined from baseline, equivalent to −10.6%. BMD of the control animals had declined, equivalent to −0.6%, and thus had remained stable. For calcium retention we observed a similar picture (Figure 1(b)). The decline from baseline was significant after 5 months until the end. Compared to the controls, there was a trend for lower values at *t*5 and *t*10 which became significant at *t*15, when values of the control group had returned to initial values. The urinary phosphorus excretion increased significantly over time compared to stable values in the control group (Figure 1(c)). In the deficient animals, osteoid surface was 55% higher than in controls at *t*15 (*P* < 0.05; Figure 2(a)). At the same time most other bone values were slightly lower in deficient animals compared to controls (Table 4). There was a trend for lower bone wet and dry weights and slightly higher fat content and slightly lower ash weight and ultimate strain (Figures 2(b) and 2(c)). OV/TV, ES/BS, Oc.S/BS, and N.Oc/T.Ar did not differ between groups (not shown).

### 3.2. Vitamin D Status and Plasma and Urinary Parameters of Bone Metabolism

**Figure 3:**
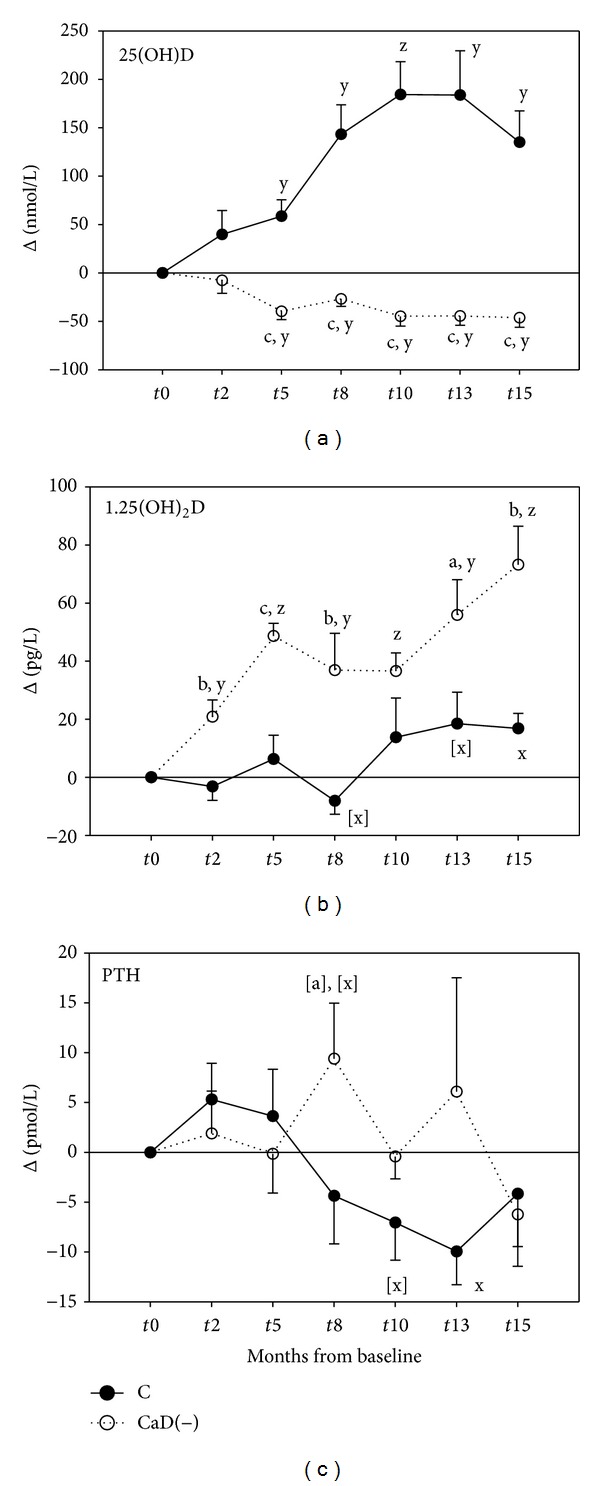
Effects of calcium and vitamin D deficiency [CaD(−); dotted line] on plasma concentrations of 25-dihydroxyvitamin D [25(OH)D], (a), 1,25-dihydroxyvitamin D [1,25(OH)_2_D], (b), and parathyroid hormone (PTH, (c)) compared with a control group (C; solid line). Values are changes from baseline *t*0, with baseline values assigned to zero. Means and SEM, *n* = 8–10. For baseline values see Table 1. Significantly different from controls at a given time: ^[a]^ *P* ≤ 0.1; ^a^ *P* ≤ 0.05; ^b^ *P* ≤ 0.01; ^c^ *P* ≤ 0.001. Significantly different from baseline: ^[x]^ *P* ≤ 0.1; ^x^ *P* ≤ 0.05; ^y^ *P* ≤ 0.01; ^z^ *P* ≤ 0.001.

When animals on our standard diet for minipigs with 2000 IU vitamin D/kg (supplying 29 *μ*g/d) were assigned to the control group [C] getting the reference diet with 6,500 IU vitamin D/kg (supplying 60 *μ*g/d) at *t*0, their plasma 25(OH)D concentrations increased significantly to reach a plateau at *t*10 (Figure 3(a)), but without augmenting BMD. In the animals on the deficient diet [CaD(−)], the plasma 25(OH)D concentration declined significantly and went along with a decrease in BMD. At *t*15 25(OH)D concentrations were 13.9 ± 2.8 nmol/L compared to 206 ± 33.8 nmol/L in the control group. In contrast to this 1,25(OH)_2_D levels increased in the deficient group from the beginning and were significantly higher than in the controls most of the time (Figure 3(b)), whose values had slightly increased over the second time period. In the deficient group plasma PTH concentrations tended to be higher than in the control group between *t*8 and *t*13 (Figure 3(c)). In that time period their values slightly increased while those of the control group slightly decreased. Plasma calcium declined and was significantly lower than in the controls after 15 months (Table 2). Urinary DPD slightly declined with time in the control group, but without being significantly different from the deficient group.

### 3.3. Mineral Absorption

After 5 months and until the end of the experiment, the fecal calcium excretion was significantly lower in the deficient group compared to the controls (Table 3) because of the low calcium content of that diet, amplified by the lack of vitamin D. These animals were in a negative calcium balance and had significantly lower values for calcium retention (Figure 1). Phosphorus intake (17.1 g/7d) was the same in both groups. Fecal phosphorus was highly variable in each group but did not differ between groups (Table 3).

## 4. Discussion

Of the many disorders that lead to rickets/osteomalacia, vitamin D deficiency and/or calcium insufficiency or its malabsorption are the most common [1, 21]. The dose effect of vitamin D from food on plasma concentrations of 25(OH)D and the dose effect of plasma 25(OH)D on bone health is still a matter of debate. Until recently the recommended daily intake (RDI) of vitamin D varied from 5 to 15 *μ*g/d (20 *μ*g/d for older adults) in the United States and parts of Europe but was recently increased for adults to 15 *μ*g/d [1] or 20 *μ*g/d [22] or is still limited to small children and the aged [23]. These doses did not take into account the pleiotropic effects of vitamin D [5, 24]. There are still open questions concerning vitamin D and calcium mediated effects on bone metabolism but also on extraskeletal issues like immunity, cardiovascular system, cancerogenesis, and central nervous system [1, 5]. Often effects and mechanisms can be more easily studied in animal models than in humans.

### 4.1. Animal Model

The pig and minipig have long been known as models for biomedical and nutritional research [25–27]. The pig, like the human, is omnivorous and, having evolved like man with significant exposure to sunlight, photo-chemically converts 7-dehydrocholesterol to cholecalciferol. The rat, in contrast, is nocturnal, with ergocalciferol its main source of vitamin D, and therefore might be less well suited for the study of vitamin D metabolism [28]. Also, because of their size, minipigs can be utilized in routine apparatus for osteodensitometry to study bone health [11, 13]. Minipigs, unlike rats or sheep, develop glucocorticosteroid-induced osteoporosis (GIO) without the need for simultaneous ovariectomy [13]. We have shown earlier that GIO can be monitored by measurement of bone mineral density [11, 13] and is accompanied by changes in cartilage matrix [29] and bone tissue vascularisation [30]. 

Basal plasma 25(OH)D concentrations in the minipigs were 60 nmol/L and 75 nmol/L and thus were higher than concentrations in young men with very low vitamin D intakes of 200 IU/day but were comparable after the men had been supplemented with 1,800 IU/day. This amount refers to a total of 25 IU vitamin D/kg body wt/d compared with the 35 IU vitamin D/kg body wt/day in the minipigs. In young growing minipiglets plasma 25(OH)D concentrations of 340 nmol/L were observed when animals were on semisynthetic diets containing 8 g calcium/kg diet and 6,500 IU/kg diet vitamin D_3_, whereas their levels were 40 nmol/L after they had consumed the same diet but devoid of vitamin D for 8 weeks [26]. In intact and ovariectomized adult domestic pigs on conventional feed, 25(OH)D concentrations were between 180 and 250 nmol/L [31]. In a study on the safety of oral 25(OH)D in growing pigs a wide range of plasma 25(OH)D was achieved without affecting numerous biological variables adversely [32]. Obviously high vitamin D supplies or comparatively high plasma 25(OH)D concentrations do not arouse toxicity or induce adverse effects on a large number of variables including those of mineral metabolism. In humans up to 10,000 IU/d of vitamin D_3_ were assumed to pose no risk of toxicity [33].

### 4.2. Modulation of Vitamin D Status—Control

When the control minipigs were switched from their 580 g/d standard diet to the 370 g/d reference diet (at *t*0), their average daily intake of vitamin D_3_ increased from 29 *μ*g/d (1,160 IU/d; 0.88 *μ*g/kg BW) to 60 *μ*g/d (2,400 IU/d; 1.83 *μ*g/kg BW) due to the different vitamin D_3_ contents and amounts of feed supplied. At the same time their mean baseline plasma 25(OH)D concentration of 75 nmol/L increased significantly within five months, with final values of 206 nmol/L at *t*15. 

Accordingly, the relation between increase of plasma 25(OH)D (131 nmol/L) and vitamin D intake or supplement (31 *μ*g/d), also calculated as the efficacy or efficiency of dietary vitamin D on vitamin D status, was approximately 4.2 nmol/L per *μ*g/d. In human studies, these values ranging between 0.6 and 2.9 nmol/L per *μ*g/d were reported [34–38]. For example, when food was fortified over a range of 3–25 *μ*g/d vitamin D, the plasma 25(OH)D concentration rose over a range of 14 and 35 nmol/L [37]. In elderly people, a daily intake of bread for 12 months fortified with 320 mg calcium and 125 *μ*g vitamin D (5000 IU) equivalent to 1.7 *μ*g/kg body weight and thus comparable to our supplemented animals increased their plasma 25(OH)D from 28 to 125 nmol/L, representing a relation of 0.8 nmol/L per *μ*g/d [36]. The efficiency values from several human studies were thus lower than those in minipigs. The reason may be a higher amount of ingested and thus absorbed vitamin D due to 100% compliance in this trial, unlike in human studies. In addition, a higher hepatic conversion to 25(OH)D in the minipigs might be assumed because of their younger biological age compared to elderly subjects. 

Nearly 40 *μ*g of vitamin D/d (1,600 IU/d; 0.51 *μ*g/kg BW) seems to ensure winter serum 25(OH)D concentrations higher than 80 nmol/L in 97.5% of the study population [34]. Participants in an Antarctic expedition were supplemented with 10, 25, or 50 *μ*g/d (0.11, 0.28, or 0.55 *μ*g/kg BW). Their plasma 25(OH)D baseline concentration of 40 nmol/L increased to 57, 63, and 71 nmol/L, respectively [39], whereas in individuals without supplement plasma 25(OH)D concentrations remained at baseline. In these studies [34, 39] the plasma 25(OH)D level after supplementation was similar to the baseline level in the minipigs (about 75 nmol/L) when getting the standard diet but was achieved with less vitamin D/kg BW, possibly because of summertime dermal synthesis and resultant higher body stores. In minipigs concentrations after supplementation stabilized around 200 nmol/L, and such high values have also been reported for subjects at the end of the summer period after having had extended outdoor activities [40]. Since some years ago there is increasing agreement on a higher upper tolerable level of vitamin D intake without reaching toxicity and of higher plasma values of 25(OH)D without having adverse physiological effects [33, 40–43]. This is also expressed in the elevation of the tolerable upper intake level (UL) of 2000 IU/d up to 4000 IU/d by the Institute of Medicine [1]. The supply with vitamin D of 2,400 IU/d in this trial remained below the upper tolerable level, even though the efficiency between vitamin D supply and plasma levels of 25(OH)D may be somewhat higher in young adult minipigs compared to aged adult humans. The plasma levels approximated a steady state after 15 months of supplementation, which is below the assumed level of toxicity of 500 nmol/L [42, 43]. This is in accordance with normocalcemia during intervention and lack of any other signs of toxicity in the animals. In order to attain mean 25(OH)D levels of about 50 nmol/L in the minipigs, about 23 ng/d (920 IU/D) might be sufficient, supposed that the calculated efficacy of 4.2 nmol/L per *μ*g/d is applicable at this lower scale of plasma values. The actual vitamin D demand might even be less, because a higher efficacy can be assumed at lower baseline values for 25(OH)D [41].

### 4.3. Modulation of Vitamin D Status—Deprivation

Vitamin D insufficiency or mild deficiency is characterized by 25(OH)D plasma concentrations less than 50 nmol/L, whereas moderate deficiency is defined by levels less than 25 nmol/L. In severe vitamin D deficiency, the mean value is lower than 10.6 nmol/L [44]. Accordingly the animals of the experimental group developed mild deficiency within five months and moderate deficiency within 10 months when mean plasma concentrations were between 15 and 20 nmol/L. Whether 25(OH)D and 1,25(OH)_2_D plasma concentrations vary in the same direction or affect each other and how their levels influence calcium absorption and BMC is not yet well established [45, 46]. In untreated nutritional osteomalacia with low levels of 25(OH)D, 1,25(OH)_2_D concentrations have been found normal [45]. In another study both vitamin D metabolites varied into the same direction when 25(OH)D concentrations were above 41 nmol/L but varied inversely at lower concentrations [46]. Thus, the direction of association may depend on the vitamin D status. It is generally assumed that the major effect of vitamin D on bone is mediated by 1,25(OH)_2_D, although 25(OH)D may have a direct effect on bone [46]. We regard the low calcium content of the deficient diet as the primary cause of the rise of plasma 1,25(OH)_2_D in our study, because calcium deficiency is a significant stimulator of plasma 1,25(OH)_2_D, and because the rise had occurred already after 2 months and thus before the descent of 25(OH)D became obvious (Figure 3). The causes of the apparent divergence of 25(OH)D and 1,25(OH)_2_D levels may be clarified in future experiments when animals on a vitamin D deficient and calcium deficient diet are compared with those on a vitamin D deficient diet on adequate supply of calcium or with those on a diet with normal vitamin D levels but low in calcium. This may be most interesting by enabling differentiation of 25(OH)D and 1,25(OH)_2_D effects *in vivo* in this minipig model which would be difficult to achieve in humans. Nevertheless, assuming one hundredth potency of 25(OH)D compared to 1,25(OH)_2_D, the D_3_ hormonal activity of both compounds taken together still showed a decrease from about 0.69 nmol/L at baseline to 0.42 nmol/L at *t*15. In the control group, this value increased from 0.85 nmol/L at baseline to 2.21 nmol/L at *t*15. 

*Osteomalacia*. In nutritional osteomalacia, defective bone mineralization leads to low BMD that might be similar to that in patients with osteoporosis [2, 8] and to a marked increase in nonmineralized bone matrix assessed histologically as osteoid volume or surface [21]. At the end of this experiment the minipigs on the deficient diet had developed hypovitaminosis D and lost bone mineral significantly according to longitudinal QCT-BMD. This loss was comparable to that in animals with GIO after 15 months on glucocorticosteroid treatment [13], although the etiologies are quite different. Their osteoid surface was significantly higher (+55%), like in the human disease [8], indicating that mild to moderate osteomalacia had developed within 15 months. BMD of minipigs is rather high [11, 13, 26] and in adult animals is about twice the value compared with human subjects [11]. This principal difference derogates a direct comparison of bone loss values between humans and minipigs. Minipigs are phenotypically more heterogenous than rodents and thus more animals are required to get the same statistical significance. The failure to reach significance for most cross-sectional differences is owed to the small number of animals, which was yet sufficient for most parameters in longitudinal comparison when paired testing achieved higher sensitivity. This is well illustrated in case of BMD (longitudinally, change of values, significant, Figure 1; cross-sectionally, actual values, nonsignificant, Table 4). The present data, however, permit calculation of adequate sample size in future experiments. 

In contrast to animals with GIO whose urinary phosphorus and BAP had decreased, 25(OH)D had increased, and 1,25(OH)_2_D was unchanged [13], these biochemical variables had increased (urinary phosphorus, 1,25(OH)_2_D), decreased (25(OH)D), or were unchanged (BAP) in the osteomalacic animals. Based on these biochemical variables nutritional osteomalacia can be discriminated from GIO in this large animal model despite similar bone loss. 

Rickets in children may result from low calcium intakes even in the face of normal vitamin D status [1, 47]. In that situation body stores of vitamin D must be adequate [48] because the rise in 1,25(OH)_2_D, when dietary calcium is low, aggravates an increase in 25(OH)D catabolism [49]. This in turn is the basis for the need to accessorily increase vitamin D intake in children with calcium deficiency rickets [50] and may be the need to accessorily increase vitamin D intake in adults on chronically low calcium intakes. 

Diagnosis of osteomalacia with the help of routine serum biochemical measures as predictors is not always clear-cut [51], because abnormal values including plasma 25(OH)D may point to osteomalacia only in its severe form but miss the less severe disease [52]. Pronounced phosphaturia, typical for rickets and osteomalacia [6, 53], has been observed in the deficient minipigs and may have aggravated BMD loss (Figure 1). In the minipigs urinary calcium excretion, plasma PTH, and markers of bone turnover were found to have changed only slightly or nonsignificantly. This contrasts with what is typically found in patients, although with large variations [2, 21, 54, 55]. The hormonal state of the estrum cycle, which we did not assess or synchronize, could have contributed to the interindividual variations of bone markers or calcium metabolism-related hormones at the certain time points. However, it could not have differently affected the cumulative and final outcomes of the two groups, like osteoid surface, bone mineral density, and vitamin D status. The study period was a long-term observation covering 15 months and thus included several of the 21-day estrogen cycles in both groups.

 The rise in PTH was delayed and less distinct, perhaps because of only a modest degree of hypovitaminosis D. Furthermore, the rise of 1,25(OH)_2_D, as induced by the calcium insufficiency, is known to suppress PTH secretion [9]. The minipigs had a lower biological age compared with studies in the elderly or patients. Patients with a diagnosis of osteomalacia have most likely experienced nutritional or other deficiencies for many years, which may explain the more obvious clinical picture compared to the one obtained in the minipig trial.

We have to state the following limitations of the study. The assay for PTH was not specific for pigs, and thus results have to be verified in the future with porcine specific assays, even though PTH was specific enough in earlier studies to detect significantly higher values for ovariectomized pigs compared to intact controls [31]. In contrast to expectations from most observations in humans, BAP did not increase, which we cannot explain at the moment. However, BAP was specific enough to detect lower values than in control pigs after glucocorticosteroid [13] and bisphosphonate treatment [11] and to discriminate osteomalacic animals from animals with GIO [56]. Absolute values were comparable to those reported by others [57]. Therefore, comparison between and longitudinal changes within groups should be plausible in this experiment. We recognize that not all changes typically associated with osteomalacia in humans were statistically significant, because the number of animals was limited. This is valid for cross-sectional studies in particular.

In conclusion, we observed that nutritional osteomalacia and modulation of vitamin D metabolites and hypovitaminosis D can be induced and studied in minipigs when on a calcium and vitamin D deficient diet. The enlarged osteoid surface in combination with a significantly lower bone mineral density in the presence of hypovitaminosis D is characteristic of the human disease. The principal advantage of the minipig model over human studies is that the time course of the various parameters of osteomalacia and hypo- or hypervitaminosis D can be evaluated over a moderately limited experimental period under controlled conditions, avoiding the problems of noncompliance beside others.

## Figures and Tables

**Table 1:** Baseline values of age, bone mineral density (BMD), and calciotropic hormones in calcium and vitamin D deficiency osteomalacia.

	Control (*n* = 9)		CaD(−) (*n* = 10)	
	Mean	SEM	Mean	SEM
Age (mo)	30	0.4	29	0.6
BMD (mg/cm³)	450.2	15.9	452.5	10.9
25(OH)D (nmol/L)	74.8	10.8	60.2	11.4
1,25(OH)_2_D (pg/mL)	42.2	5.8	38.4	5.0
PTH (pmol/L)	18.5	2.2	19.9	3.6

Mean values and standard error of the mean. There were no significant differences between groups.

**Table 2:** Baseline values and time course of body weights and markers of calcium metabolism and bone turnover in calcium and vitamin D deficiency osteomalacia.

	Control (*n* = 9)						CaD(−) (*n* = 10)					
	*t*0		*t*8		*t*15		*t*0		*t*8		*t*15	
	Mean	SEM	Mean	SEM	Mean	SEM	Mean	SEM	Mean	SEM	Mean	SEM
Body weight (kg)	33.1	1.6	33.5	1.3	33.1	1.3	32.4	1.8	32.0	1.2	31.2	1.5
Plasma												
Ca (mg/L)	110.1	3.3	107.6	2.4	107.6	2.2	111.0	1.9	108.5	1.6	101.5	1.5^a,x^
P (mg/L)	49.7	1.9	46.7	2.7	52.7	2.1	49.5	2.4	47.7	0.8	50.1	1.3
AP (U/L)	116.9	28.9	153.5	59.6	185.0	75.2	139.1	23.4	200.7	44.1^x^	169.2	22.3
BAP (U/L)	29.6	4.6	29.6	4.2	23.3	3.2	38.0	4.9	36.1	4.6	36.0	5.8^[a]^
OPG (pmol/L)	0.9	0.1	0.6	0.1	nd		0.8	0.1	0.73	0.04^[x]^	nd	
Urine												
DPD (nmol/24 h)	139.9	25.5	116.0	12.2	84.3	12.2	120.3	2.3	128.3	16.9	81.8	7.6
DPD/Crea (nmol/mmol)	10.1	3.2	6.2	0.7	6.7	1.6	11.8	2.9	5.5	0.9^[x]^	5.5	0.9

Mean values and standard error of the mean. AP: total alkaline phosphatase; BAP: bone alkaline phosphatase, OPG: osteoprotegerin; nd: not determined; DPD: deoxypyridinoline; Crea: creatinine. Samples are 8–10. Significantly different from the value of the control group at the same time point with ^[a]^ *P* ≤ 0.1; ^a^ *P* ≤ 0.05. Significantly different from the baseline value with ^[x]^ *P* ≤ 0.1; ^x^ *P* ≤ 0.05.

**Table 3:** Baseline and final values of calcium and phosphorus balances in calcium and vitamin D deficiency osteomalacia.

	Control (*n* = 9)				CaD(−) (*n* = 10)			
	Baseline (*t*0)		Final (*t*15)		Baseline (*t*0)		Final (*t*15)	
	Mean	SEM	Mean	SEM	Mean	SEM	Mean	SEM
Ca intake (mg/7d)	15540		15540		15540		5180	
Faecal Ca (mg/7d)	13699	850	13833	1232	12558	1079	8373	2258^a,[x]^
Urinary Ca (mg/7d)	431	79	391	68	383	33	460	85
Ca absorption (mg/7d)	1841	850	1707	1232	2982	1079	−3193	2258^[a],x^
Ca retention (mg/7d)	1410	893	1316	1261	2599	1094	−3654	2231^[a],x^

P intake (mg/7d)	17094		17094		17094		17094	
Faecal P (mg/7d)	16433	1669	14050	1116	14161	1090	12336	2186
Urinary P (mg/7d)	1103	198	1196	178	1540	299	3207	545^b,x^
P absorption (mg/7d)	661	1669	3044	1116	2933	1090	4758	2186
P retention (mg/7d)	−441	1666	1848	1158	1393	991	1551	2235

Mean values and standard error of the mean. Significantly different from the value of the control group at the same time point with ^[a]^ *P* ≤ 0.1; ^a^ *P* ≤ 0.05; ^b^ *P* ≤ 0.01; ^c^ *P* ≤ 0.001. Significantly different from the baseline value with ^[x]^ *P* ≤ 0.1; ^x^ *P* ≤ 0.05.

**Table 4:** Cross-sectional effects of calcium and vitamin D deficiency osteomalacia on chemical composition, material properties, *in vivo* BMD, and morphology in bone specimen at *t*15.

	Control (*n* = 9)		CaD(−) (*n* = 10)	
	Mean	SEM	Mean	SEM
4th lumbar vertebra				
Bone wet weight (mg)^b^	594	17	550	16^[a]^
Bone dry weight (mg)^b^	350	12	318	12^[a]^
Organic matter (mg)^b^	143	7	133	5
Calcium (mg)^b^	128.7	4.1	125.1	2.9
Phosphorus (mg)^b^	59.6	2.1	57.5	1.3
Fat (mg)^c,d^	8.3	1.4	9.3	1.3
Young's modulus (MPa)	1952	145	1793	150
Breaking load (N)	1118	80	973	72
Ultimate stress (MPa)	25.3	1.8	22.0	1.6
2nd lumbar vertebra				
BMD^e^ (mg/cm^3^)	447.5	20.9	404.5	17.2
BV/TV (%)	34.0	3.1	30.7	1.8
BMD/(BV/TV)^f^ (g/mL)	1.4	0.2	1.3	0.3
Tb.Wi (*µ*m)	121.6	10.7	106.4	7.1
Tb.Sp (*µ*m)	245.4	26.0	242.5	15.0
Ob.S/BS (%)	4.0	2.1	6.4	2.8
N.Oc/BS (p.mm)	0.8	0.2	1.0	0.3

Mean values and standard error of the mean. The chemical composition and material properties were determined on the same cylindrical specimen. The 2nd lumbar vertebrae were analysed morphologically (*n* = 8–10). BV/TV: bone volume/tissue volume; BMD: bone mineral density; Tb.Wi: trabecular width; Tb.Sp: trabecular separation; Ob.S/BS: osteoblast surface/bone surface; N.Oc/BS: number of osteoclasts/bone surface. None of the differences reached statistical significance, ^[a]^ *P* ≤ 0.1. ^b^mg/cylindrical sample; ^c^mg/100 mg bone wet weight; ^d^in the last breast vertebra; ^e^ *in vivo *in L1–L3; ^f^indicates mineralisation of bone tissue.
